# Primary EBV Infection Induces an Acute Wave of Activated Antigen-Specific Cytotoxic CD4^+^ T Cells

**DOI:** 10.4049/jimmunol.1900377

**Published:** 2019-07-15

**Authors:** Benjamin J. Meckiff, Kristin Ladell, James E. McLaren, Gordon B. Ryan, Alison M. Leese, Eddie A. James, David A. Price, Heather M. Long

**Affiliations:** * Institute of Immunology and Immunotherapy, University of Birmingham, Edgbaston, Birmingham B15 2TT, United Kingdom;; † Division of Infection and Immunity, Cardiff University School of Medicine, Cardiff CF14 4XN, United Kingdom; and; ‡ Tetramer Core Laboratory, Diabetes Program, Benaroya Research Institute at Virginia Mason, Seattle, WA 98101

## Abstract

CD4^+^ T cells are essential for immune protection against viruses, yet their multiple roles remain ill-defined at the single-cell level in humans. Using HLA class II tetramers, we studied the functional properties and clonotypic architecture of EBV-specific CD4^+^ T cells in patients with infectious mononucleosis, a symptomatic manifestation of primary EBV infection, and in long-term healthy carriers of EBV. We found that primary infection elicited oligoclonal expansions of T_H_1-like EBV-specific CD4^+^ T cells armed with cytotoxic proteins that responded immediately ex vivo to challenge with EBV-infected B cells. Importantly, these acutely generated cytotoxic CD4^+^ T cells were highly activated and transcriptionally distinct from classically described cytotoxic CD4^+^ memory T cells that accumulate during other persistent viral infections, including CMV and HIV. In contrast, EBV-specific memory CD4^+^ T cells displayed increased cytokine polyfunctionality but lacked cytotoxic activity. These findings suggested an important effector role for acutely generated cytotoxic CD4^+^ T cells that could potentially be harnessed to improve the efficacy of vaccines against EBV.

## Key Points

- Primary EBV infection drives highly cytotoxic virus-specific CD4^+^ T cell responses.
- EBV-specific memory CD4^+^ T cells are polyfunctional but lack cytotoxic activity.
- Acute EBV-specific CD4-CTLs differ transcriptionally from classical memory CD4-CTLs.

## Introduction

Efficient long-term control of persistent viral infection requires the coordinated action of Ag-specific CD4^+^ and CD8^+^ T cells (1). Upon Ag encounter, naive MHC class II (MHCII)–restricted CD4^+^ T cells possess the ability to differentiate into several distinct effector subsets, reflecting their various helper roles in the immune response. After resolution of the initial challenge, small populations of circulating Ag-specific CD4^+^ T cells are retained as central memory T cells (T_CM_; CCR7^+^CD45RA^−^) or effector memory T cells (T_EM_; CCR7^−^CD45RA^−^) (2). As a consequence, the total CD4^+^ T cell pool is functionally and phenotypically heterogeneous (3). However, at the Ag-specific level, virus-induced CD4^+^ T cell responses are substantially less well defined in humans. Little is known of the clonal composition or functional diversity within individual epitope-specific populations or how virus-specific CD4^+^ T cell responses evolve from primary to persistent viral infection (4, 5).

In addition to their helper roles, it is now appreciated that some CD4^+^ T cells can acquire perforin (Perf)/granzyme B (GzmB)–mediated cytotoxic function, akin to CD8^+^ T cells (6). The ability of cytotoxic CD4^+^ T cells (CD4-CTLs) to directly kill MHCII^+^ targets expressing cognate Ag in vitro has raised significant interest among viral and tumor immunologists alike (7, 8). Such functionality is particularly valuable for viral infections or cancers occurring in cell lineages that either naturally express MHCII, or can be induced to express MHCII, for example, following infection or transformation. In vivo, CD4-CTLs have been predominantly reported in humans in the context of persistent viral infections, including CMV and HIV (8, 9), and various cancers (7, 10). These collective observations suggested a key role for chronic Ag exposure and progressive differentiation in the acquisition of cytotoxic activity by CD4-CTLs, which often display a late differentiated terminal effector memory T cell (T_EMRA_; CCR7^−^CD45RA^−^). Rare indications of CD4-CTLs in more acute settings or following vaccination exist, but the relevance of these observations remains unclear (11, 12). Induction of CD4-CTLs is now considered an important goal in the design of many next-generation vaccines (4, 13); however, greater definition of naturally protective CD4^+^ T cells at the Ag-specific and single-cell level is first required. To date, a lack of known epitopes and associated reagents for the ex vivo detection of Ag-specific CD4^+^ T cells has limited functional and clonotypic studies almost exclusively to analyses of the entire CD4^+^ T cell pool or peptide-stimulated CD4^+^ T cell populations, mostly following in vitro culture (14–19).

In this study, we focus on EBV, an orally transmitted herpesvirus that establishes lifelong infection of the memory B cell pool (20). Primary EBV infection is often asymptomatic, but some individuals present clinically with infectious mononucleosis (IM). Early diagnosis is facilitated in these cases and provides a unique opportunity to study the evolution of immune responses to viral infection in humans (21). In a previous study, we optimized EBV peptide-MHCII (pMHCII) tetramer analysis to facilitate ex vivo detection of EBV epitope-specific CD4^+^ T cell populations and demonstrated that primary infection elicits high frequencies of virus-specific CD4^+^ T cells against a broad range of Ags (22). These initial expansions are rapidly culled to leave small populations of T_CM_ and T_EM_ CD4^+^ T cells that persist throughout the chronic phase of infection. Importantly, EBV-infected memory B cells uniformly express MHCII (20), and several groups have shown that in vitro–isolated EBV-specific CD4^+^ T cells can recognize and, in some cases, kill virus-transformed B cells (23–29). If also true in vivo, such functionality would be highly advantageous against EBV infection and associated diseases.

In this study, we used pMHCII tetramers to dissect the functional and clonotypic properties of EBV-specific CD4^+^ T cells. We showed that primary EBV infection elicits oligoclonal populations of highly activated and cytotoxic EBV-specific CD4^+^ T cells, which responded directly ex vivo to EBV-transformed B cells. These acutely generated CD4-CTLs differed transcriptionally from classically defined CD4-CTLs and waned through convalescence to almost undetectable frequencies in the long-term Ag-specific memory pool. Our findings provided valuable insights into human CD4^+^ T cell immunity against EBV infection, with potential implications for the rational design of vaccines against EBV.

## Materials and Methods

### Donors and ethics

The study cohort included 14 healthy carriers and 16 patients with acute IM. All donors provided written informed consent in accordance with the Declaration of Helsinki. Convalescence was defined temporally as 6 mo after the initial diagnosis of IM. Study approval was granted by the South Birmingham Local Research Ethics Committee (14/WM/1254).

### Samples

PBMCs were separated via Ficoll-Hypaque centrifugation into RPMI 1640 medium (Sigma-Aldrich) supplemented with 8% FCS, 100 IU/ml penicillin, 100 μg/ml streptomycin, and 2 mM l-glutamine (R8). CD8^+^ T cells were depleted using anti-CD8 Dynabeads (Life Technologies), and CD4^+^ T cells were enriched using a Human CD4^+^ T Cell Enrichment Kit (STEMCELL Technologies). Unless indicated otherwise, all experiments were performed using enriched populations of CD4^+^ T cells with purities >95%.

### Flow cytometry

Total or CD8-depleted PBMCs from healthy carriers or CD4-enriched PBMCs from patients with IM were stained with optimized concentrations of peptide­–MHC class I (pMHCI) (University of Birmingham) or pMHCII tetramers (Tetramer Core Laboratory of the Benaroya Research Institute) containing epitopes from EBV or influenza virus (influenza A/New York/348/03 H1N1) appropriate for the HLA type of each donor (Table I). Cells were washed in PBS prior to pMHCI tetramer staining for 30 min or in human serum prior to pMHCII tetramer staining for 1 h. After incubation, cells were washed in PBS, and nonviable events were labeled using a LIVE/DEAD Fixable Dead Cell Stain Kit (eBioscience or Thermo Fisher Scientific). Surface markers were identified using the following directly conjugated Abs: anti-CD3 (SK7), anti-CD4 (RPA-T4), anti-CD14 (HCD14), anti-CD19 (HIB19), anti-CD38 (HIT2), anti-CD69 (FN50), anti-CCR5 (J418F1), and anti-NKG2D (1D11) (BioLegend); anti-CD8 (RPA-T8), anti-CD14 (HCD14), anti-CD19 (HIB19), anti-CD107a (H4A3), anti-CCR7 (3D12), and anti-CX_3_CR1 (2A9-1) (BD Biosciences); and anti-CD45RA (2H4LDH11LDB9) (Beckman Coulter). For detection of effector molecules, cells were fixed/permeabilized using paraformaldehyde/saponin and stained with the following directly conjugated Abs: anti–GM-CSF (BVD2-21C11), anti-GzmB (GB11), anti–IFN-γ (4SB3), anti–IL-2 (MQ1-17H12), anti–IL-4 (8D4-8), anti–IL-10 (JES3-9D7), anti–IL-21 (3A3-N2), and anti–TNF-α (Mab11) (BioLegend); and anti-Perf (δg9) (eBioscience). For detection of transcription factors, cells were fixed/permeabilized using a Transcription Factor Staining Buffer Set (eBioscience) and stained with the following directly conjugated Abs: anti–T-bet (4B10), anti-FOXP3 (236A/E7), and anti-Eomes (WD1928) (eBioscience); and anti-Gata3 (L50-823) and anti-Hobit (Sanquin-Hobit/1) (BD Biosciences). All Ab reagents were pretitrated for optimal performance. Data were acquired using an LSR II or a Fortessa flow cytometer (BD Biosciences) and processed using Kaluza Analysis Software (Beckman Coulter).

### Cell stimulation and functional profiles

CD8-depleted PBMCs from DR7^+^ healthy carriers were stimulated with the EBNA2_276–295_ peptide at a concentration 0.005 μg/ml for 4 h in the presence of brefeldin A (BD Biosciences). EBV-transformed autologous B lymphoblastoid cell lines (LCLs) were generated as described previously (30). Bulk PBMCs from healthy carriers or patients with IM were stimulated with autologous LCLs at a ratio of 1:1 for 16 h in the presence of brefeldin A, monensin, and anti–CD107a-FITC (H4A3) (BD Biosciences). Stimulated cells were washed twice in R8 and processed as described above prior to flow cytometric analysis of CD107a, IFN-γ, IL-2, and TNF-α.

### TCR repertoire analysis

Viable EBV-specific tetramer^+^ CD4^+^ T cell populations were sorted at >98% purity directly into RNAlater (Thermo Fisher Scientific) using a custom-modified FACSAria II flow cytometer equipped with DIVA software version 8.0.1 (BD Biosciences). Unbiased amplification of all expressed *TRB* gene rearrangements was conducted using a template-switch–anchored RT-PCR with a 3′ C region primer (31). Amplicons were subcloned, sampled, sequenced, and analyzed as described previously (32). Gene use was assigned using the International ImMunoGeneTics nomenclature (33). All functional TCR sequences were deposited online at VDJdb (34). Expression of defined TCR Vβ segments on the surface of EBV-specific CD4^+^ T cells was assessed using a TCR Vβ Repertoire Kit (Beckman Coulter).

### Statistics

Statistical comparisons were performed using the Mann–Whitney *U* test, the Spearman rank test, or an unpaired Student *t* test with Welch correction implemented via Prism 7 (GraphPad). The functional profiles of LCL-responsive EBV-specific CD4^+^ T cells were compared using Funky Cells and SPICE software version 5.32 (35).

## Results

### Ex vivo functional properties of EBV-specific memory CD4^+^ T cells

**FIGURE 1.**
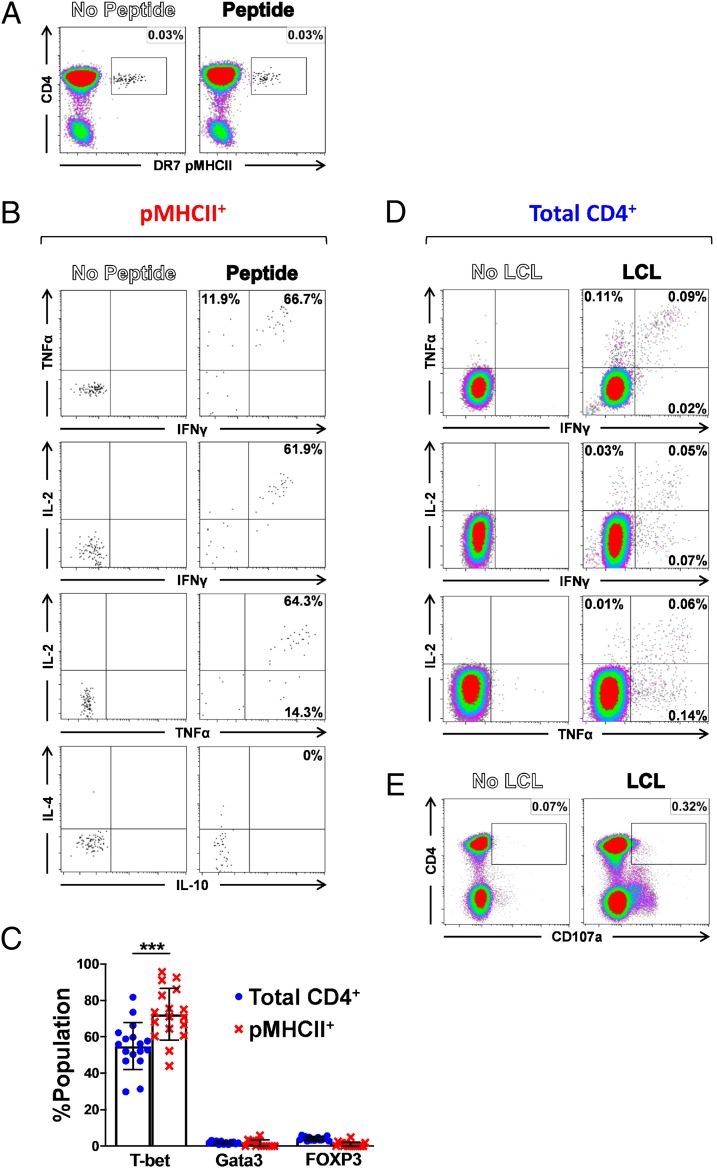
EBV-specific memory CD4^+^ T cells are polyfunctional. (**A** and **B**) CD4-enriched PBMCs from a representative healthy carrier were either unmanipulated or stimulated with EBNA2_276–295_ peptide, stained with DR7/EBNA2_276–295_ tetramer, and analyzed by flow cytometry for intracellular expression of IFN-γ, TNF-α, and IL-2. (A) Representative DR7/EBNA2_276–295_ tetramer staining of unmanipulated (left panel) and peptide-stimulated PBMCs (right panel). (B) Cytokine production in unmanipulated (left column) and peptide-stimulated DR7/EBNA2_276–295_ tetramer^+^ CD4^+^ T cells (right column). (**C**) Summary of transcription factor expression in unstimulated total CD4^+^ (blue) and EBV-specific pMHCII tetramer^+^ CD4^+^ T cell populations (red) from healthy carriers (T-bet, *n* = 17; GATA3, *n* = 13; FoxP3, *n* = 17). The corresponding pMHCII tetramers are listed in Table I. (**D** and **E**) PBMCs from healthy carriers were either unmanipulated or stimulated with autologous LCLs and analyzed by flow cytometry for intracellular expression of IFN-γ, TNF-α, and IL-2 (D), and surface mobilization of CD107a (E). Plots are gated on CD4^+^ T cells. Results are representative of at least three independent experiments with four donors. Error bars indicate means ± SD. Significance was determined by unpaired Student *t* test with Welch correction (c). ***p < 0.001.

To profile the functional capabilities of EBV-specific memory CD4^+^ T cells in healthy virus carriers directly ex vivo, we employed a panel of pMHCII tetramers (Table I) optimized in our previous work (22). These reagents were used in conjunction with intracellular flow cytometry to assess cytokine production in EBV-specific CD4^+^ T cells following cognate peptide stimulation. The gating strategy for data analysis (Supplemental Fig. 1) was consistent with the recommendations of the Cancer Immunotherapy Consortium (36). Careful experimental optimization was performed to maintain sufficient TCR expression on the surface of peptide-responsive CD4^+^ T cells to allow subsequent staining with relevant pMHCII tetramers (Fig. 1A). As illustrated for a representative healthy carrier, the majority of HLA DRB1*07:01 (DR7)–restricted EBNA2_276–295_–specific CD4^+^ T cells produced the effector cytokines IFN-γ, TNF-α, and IL-2 (Fig. 1B). However, 21.4% of pMHCII tetramer^+^ cells made none of these cytokines, most likely reflecting those residing in the T_CM_ component, which is less responsive ex vivo (2, 16, 22). Interestingly, TNF-α was the most predominantly produced cytokine in healthy carriers, made in isolation by 11.9% and in total by 78.6% of DR7/EBNA2_276–295_–specific CD4^+^ T cells. All IFN-γ^+^ and/or IL-2^+^ cells simultaneously produced TNF-α. There was no detectable production of IL-4, IL-10, IL-21, or GM-CSF among DR7/EBNA2_276–295_–specific CD4^+^ T cells (data not shown and Fig. 1B). In line with these findings, the transcription factor T-bet was expressed in the vast majority of DR7/EBNA2_276–295_–specific CD4^+^ T cells (mean 72.4%, *n* = 17) (Fig. 1C). Accordingly, EBV-specific CD4^+^ T cells in healthy carriers displayed a T_H_1-like response to Ag encounter directly ex vivo, consistent with a typical distribution of T_EM_ and T_CM_ memory phenotypes (22).

**Table I.** pMHCI and pMHCII tetramers

MHC Class	Pathogen	Virus Phase	Protein	Coordinates	Epitope	MHC Restriction
MHCI	EBV	Lytic	BMLF1	259–267	GLCTLVAML	A2:01
MHCII	EBV	Latent	EBNA1 (E1)	474–493	SNPKFENIAEGLRVLLARSH	DRB5*01:01 (DR51)
MHCII	EBV	Latent	EBNA2 (E2)	276–295	PRSPTVFYNIPPMPLPPSQL	DRB1*07:01 (DR7)
MHCII	EBV	Latent	EBNA2 (E2)	279–295	PRSPTVFYNIPPMPLPPSQL	DRB3*02:02 (DR52b)
MHCII	EBV	Latent	EBNA2 (E2)	301–320	PAQPPPGVINDQQLHHLPSG	DRB1*03:01 (DR17)
MHCII	EBV	Lytic	BMRF1 (BM)	136–150	VKLTMEYDDKVSKSH	DRB1*03:01 (DR17)
MHCII	EBV	Lytic	BaRF1 (Ba)	185–199	SRDELLHTRAASLLY	DRB1*07:01 (DR7)
MHCII	Influenza	N/A	Matrix protein 1 (M1)	43–59	MEWLKTRPILSPLTKGI	DRB1*07:01 (DR7)

**FIGURE 2.**
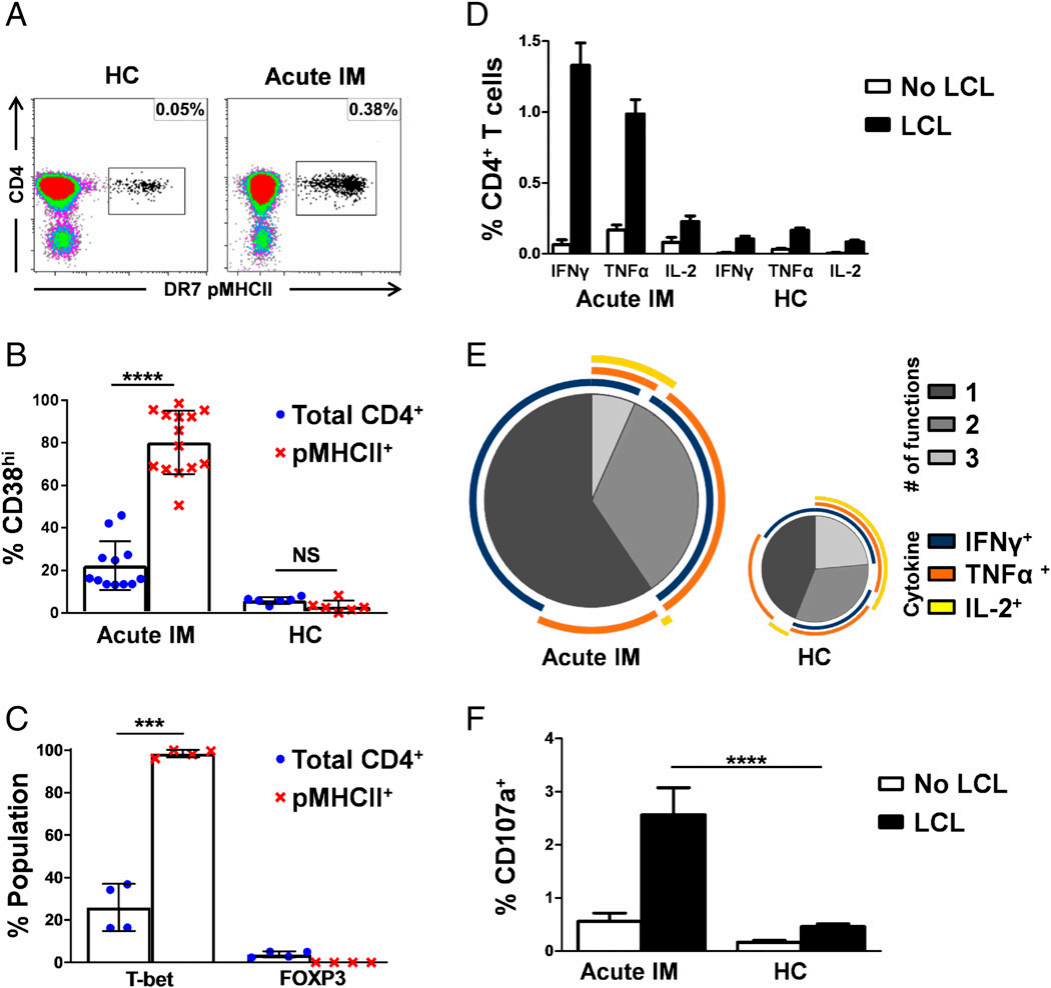
Acute IM drives expanded populations of polyfunctional EBV-specific CD4^+^ T cells. (**A**–**C**) CD4-enriched PBMCs from patients with IM and healthy carriers were stained with the relevant pMHCII tetramers (Table I) (A) and analyzed for surface expression of CD38 (*n* = 13 for acute IM and *n* = 6 for healthy carriers[HC]) (B) and intracellular expression of T-bet and FoxP3 in patients with IM (*n* = 4) (C) by flow cytometry. Blue, total CD4^+^ T cells; red, EBV-specific pMHCII tetramer^+^ CD4^+^ T cells. (**D**–**F**) PBMCs from patients with IM and HC were either unmanipulated (unfilled) or stimulated with autologous LCLs (filled) and analyzed by flow cytometry for intracellular expression of IFN-γ, TNF-α, and IL-2 (D and E) and surface mobilization of CD107a (F). The summary graphs (D and F) depict results from eight independent experiments with a minimum of four donors. (E) SPICE plots illustrating the functional profiles of CD4^+^ T cells from patients with IM and HC responding to autologous LCLs. Image sizes are scaled to depict the overall numbers of responding cells. Each pie chart segment displays the fraction of responding cells producing the number of cytokines indicated in the key. Arcs show the distribution of each individual function. Error bars indicate means ± SD. Significance was determined by unpaired Student *t* test with Welch correction (b and c) or the Mann–Whitney U test (f). ***p < 0.001, ****p < 0.0001.

In further experiments, we tested the ability of EBV-specific memory CD4^+^ T cells to directly recognize virus-infected cells ex vivo, an observation previously limited to cultured cells in the in vitro setting (23–29). As shown for the representative healthy carrier in Fig. 1D (and in the concatenated data from all donors in Fig. 2D) low frequencies of CD4^+^ T cells produced effector cytokines after overnight stimulation of PBMCs with autologous EBV-transformed B LCLs. These responses were similarly T_H_1 polarized; however, higher proportions of LCL-stimulated CD4^+^ T cells produced IFN-γ, TNF-α, or IL-2 in isolation compared with peptide-stimulated CD4^+^ T cells. This may reflect lower epitope densities in the context of natural Ag presentation or functional heterogeneity among different populations of epitope-specific CD4^+^ T cells. Small fractions of CD4^+^ T cells also mobilized CD107a, indicating degranulation following LCL stimulation (Fig. 1E).

### Expansion of polyfunctional EBV-specific CD4^+^ T cells during primary EBV infection

We next used pMHCII tetramers to investigate the functional profile of EBV-specific CD4^+^ T cells induced by primary infection, in which we have previously shown expansions of effector CD4^+^ T cells (CD45RA^+^, CCR7^−^, CD62L^−^) specific for a broad range of EBV Ags, with frequencies up to 10-fold greater than those detected in healthy carriers (22). In line with these earlier findings, the present cohort of patients with acute IM similarly had higher frequencies of EBV-specific CD4^+^ T cells (Fig. 2A), and the majority of these cells expressed the activation marker CD38 (CD38^hi^) (Fig. 2B). Moreover, T-bet expression was almost universal among EBV-specific CD4^+^ T cells in the setting of IM (mean 98.5%), significantly exceeding T-bet expression frequencies among total CD4^+^ T cells in the same donors (mean 26.0%, *p* < 0.001; Fig. 2C) and EBV-specific memory CD4^+^ T cells in healthy carriers (mean 72.4%, *p* < 0.001; Fig. 1C). Interestingly, FOXP3 expression was almost undetectable among EBV-specific CD4^+^ T cells in donors with either acute (Fig. 2C) or persistent infection (Fig. 1C).

In light of these findings, we hypothesized that EBV-specific CD4^+^ T cells in patients with IM might respond more efficiently ex vivo to cognate Ag encounter. Accordingly, substantially higher frequencies of responsive CD4^+^ T cells were detected in patients with IM compared with healthy carriers after overnight stimulation of PBMCs with autologous LCL (Fig. 2D). Furthermore, the responding CD4^+^ T cells had different functional profiles at the two stages of infection. In acute IM, smaller fractions of CD4^+^ T cells were polyfunctional (IFN-γ^+^, TNF-α^+^, IL-2^+^) than in healthy carriers (mean 6.5% versus mean 23.2%, respectively, *p* < 0.05; Fig. 2E), in part reflecting more frequent production of IL-2 during persistent infection (Fig. 2E). In addition, IFN-γ was the dominant cytokine produced by responsive CD4^+^ T cells in patients with IM (mean 81.9% for IFN-γ versus mean 54.0% for TNF-α, *p* < 0.05; Fig. 2E), whereas TNF-α was the dominant cytokine produced by responsive CD4^+^ T cells in healthy carriers (mean 75% for TNF-α versus mean 64.8% for IFN-γ; Fig. 2E). Moreover, higher frequencies of LCL-stimulated CD4^+^ T cells mobilized CD107a in patients with IM compared with healthy carriers (mean 2.6% versus mean <0.5%, respectively, *p* < 0.0001; Fig. 2E), indicating higher levels of degranulation in response to EBV-infected B cells in acute infection. Interestingly, in acute infection, degranulation generally occurred without coincident detectable production of IL-2.

### Expression of cytotoxic proteins in EBV-specific CD4^+^ T cells

**FIGURE 3.**
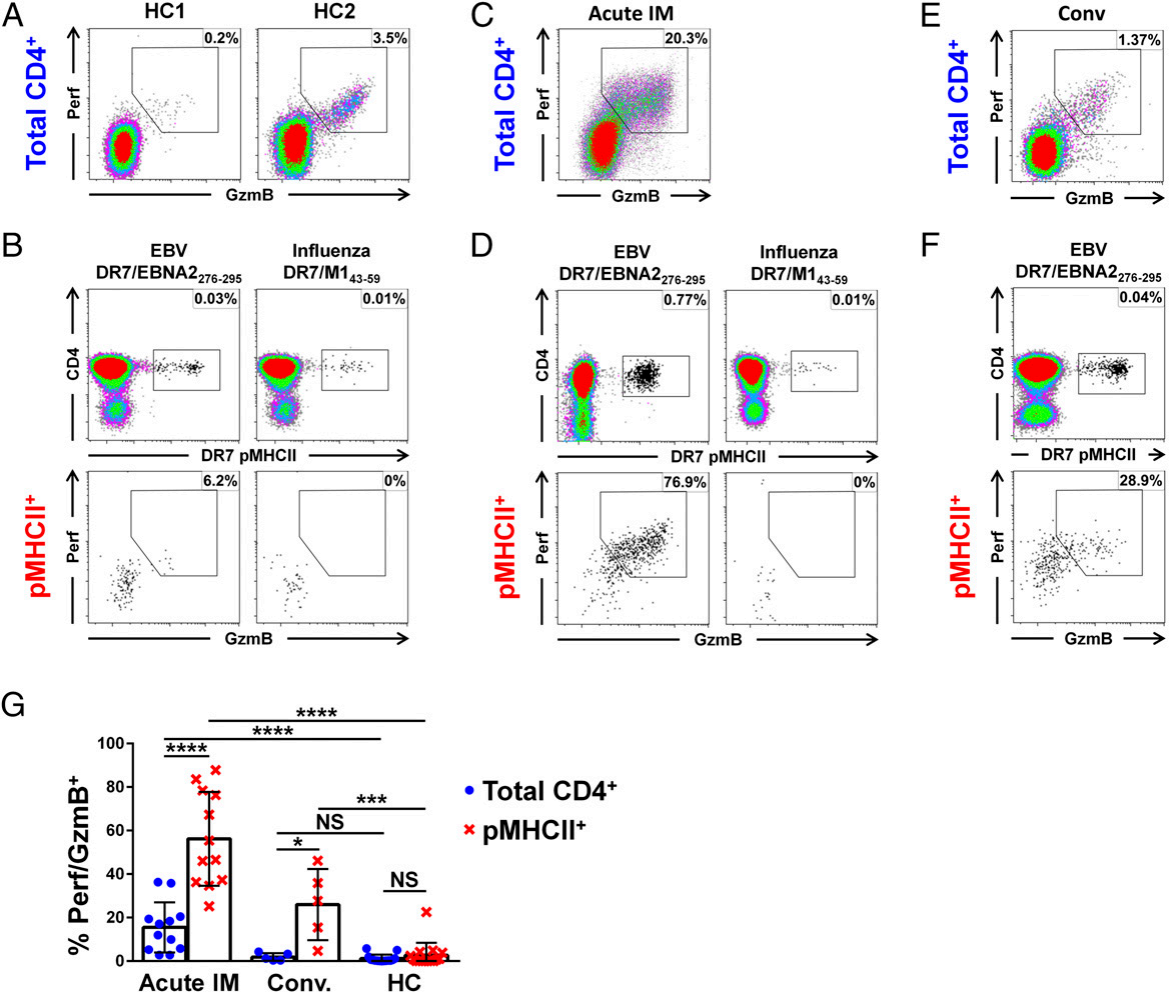
EBV-specific CD4^+^ T cells express cytotoxic proteins in patients with IM. (**A**–**G**) CD4-enriched PBMCs from healthy carriers (HC), patients with acute IM and patients with convalescent IM were stained with the relevant pMHCII tetramers (Table I) and analyzed by flow cytometry for intracellular expression of Perf/GzmB. (A) Perf/GzmB expression in the total CD4^+^ T cell pool shown for two representative HC. (B) Representative DR7/EBNA2_276–295_ and DR7/M1_43–59_ tetramer stains (top panels) and Perf/GzmB expression among pMHCII tetramer^+^ CD4^+^ T cells (bottom panels) shown for a DR7^+^ HC. (C) Perf/GzmB expression in the total CD4^+^ T cell pool shown for a representative patient with acute IM. (D) Representative DR7/EBNA2_276–295_ and DR7/M1_43–59_ tetramer stains (top panels) and Perf/GzmB expression among pMHCII tetramer^+^ CD4^+^ T cells (bottom panels) shown for a DR7^+^ patient with acute IM. (E) Perf/GzmB in the total CD4^+^ T cell pool shown for a representative patient during convalescence. (F) Representative DR7/EBNA2_276–295_ tetramer stain (top panels) and Perf/GzmB expression among pMHCII tetramer^+^ CD4^+^ T cells (bottom panels) shown for a DR7^+^ patient during convalescence. (G) Summary of Perf/GzmB expression in the total CD4^+^ (blue) and EBV-specific pMHCII tetramer^+^ CD4^+^ T cell populations (red) from patients with acute IM (*n* = 12), patients with convalescent IM (*n* = 5), and HC (*n* = 15). Error bars indicate means ± SD. Significance was determined by unpaired Student *t* test with Welch correction between total CD4^+^ and pMHCII^+^ populations and the Mann–Whitney U test for comparison between donor groups (h). *p < 0.05, ***p < 0.001, ****p < 0.0001. NS, not significant.

Degranulation is necessary but not sufficient for cytotoxic activity. Therefore, to further investigate cytotoxic function, we assessed the expression of Perf and GzmB in pMHCII tetramer^+^ CD4^+^ T cells directly ex vivo without prior in vitro stimulation. To calibrate our measurements, we analyzed an EBV-specific memory CD8^+^ T response against the HLA-A*02:01 (A2)–restricted epitope BMLF1_259–267_, which is known to express Perf and GzmB in vivo (37). As expected, Perf/GzmB were expressed by 36.5% of all CD8^+^ T cells (Supplemental Fig. 2a) and 69.9% of A2/BMLF1_259–267_–specific CD8^+^ T cells in a representative healthy carrier (Supplemental Fig. 2b). In contrast, relatively low percentages of total CD4^+^ T cells (typically <5%) expressed Perf/GzmB in healthy carriers (Fig. 3A, 3G), and this was similarly the case for the EBV-specific memory CD4^+^ T cells. In the example healthy carrier shown, with a DR7/EBNA2_276–295_–specific population comprising 0.03% of all CD4^+^ T cells, only 6.2% of the tetramer^+^ cells expressed Perf/GzmB (Fig. 3B, left panels). Interestingly, no Perf/GzmB^+^ events were detected in a coexistent DR7-restricted memory CD4^+^ T cell population specific for the influenza virus A matrix protein 1 epitope M1_43–59_ (38) analyzed at the same time point (Fig. 3B, right panels).

In contrast, Perf/GzmB were expressed by a greater frequency of total CD4^+^ T cells in patients with IM compared with healthy carriers (Fig. 3C, 3G). Furthermore, in the representative IM patient shown, in whom 0.77% of all CD4^+^ T cells were specific for DR7/EBNA2_276–295_, a remarkable 76.9% of the pMHCII tetramer^+^ cells expressed Perf/GzmB (Fig. 3D, left panels). In contrast, no Perf/GzmB^+^ events were detected among a coexisting influenza M1_43–59_–specific CD4^+^ T cells in this donor (Fig. 3D, right panels), or any other patients with IM (*n* = 5), excluding the possibility of bystander CD4^+^ T cell activation. Five of the patients with acute IM donated further blood samples 6 mo after the initial diagnosis of IM, enabling analysis of Perf/GzmB expression in EBV-specific CD4^+^ T cells during convalescence. Interestingly, despite contraction of both the DR7/EBNA2_276–295_–specific CD4^+^ T cell population (Fig. 3F, top plot) and Perf/GzmB expression in the total CD4^+^ T cell pool (Fig. 3E) to frequencies typical of long-term virus carriage (Fig. 3A, 3B), Perf/GzmB expression remained elevated among the pMHCII tetramer^+^ cells (Fig. 3F, bottom plot).

The concatenated data for all patients with acute IM (*n* = 12), patients with convalescent IM (*n* = 5), and healthy carriers (*n* = 15) are shown in Fig. 3G. Perf/GzmB expression in EBV-specific CD4^+^ T cells was highest in patients with acute IM but was variable between donors (25.2–87.8%), likely reflecting differences in time from symptomatic presentation to diagnosis (typically 10–21 d after disease onset). However, in all cases, much higher frequencies of EBV-specific CD4^+^ T cells expressed Perf/GzmB in patients with acute IM compared with healthy carriers (mean 56.2% versus mean 2.7%, respectively, *p* < 0.0001). Notably, Perf/GzmB expression was also significantly raised among total CD4^+^ T cells during acute infection (mean 15.5% for patients with IM versus mean 1.2% for healthy carriers, *p* < 0.0001). This suggested that the combined expansions of the broadly targeted EBV-specific CD4^+^ T cell responses in acute IM were large enough to influence Perf/GzmB expression in the total CD4^+^ T cell pool. In the patients with acute IM sampled again during convalescence, Perf/GzmB expression frequencies in the total CD4^+^ T cell pool had largely dropped to levels equivalent to those observed in healthy carriers. However, expression of the cytotoxic proteins remained at least partially elevated in some EBV-specific populations (mean 26.0% for patients with convalescent IM versus mean 2.7% for healthy carriers, *p* < 0.001). Together, these data show that CD4-CTLs are a major component of virus-specific CD4^+^ T cell responses induced by primary EBV infection but not of those maintained in long-term memory.

### Expression of cytotoxicity-associated proteins in EBV-specific CD4^+^ T cells

**FIGURE 4.**
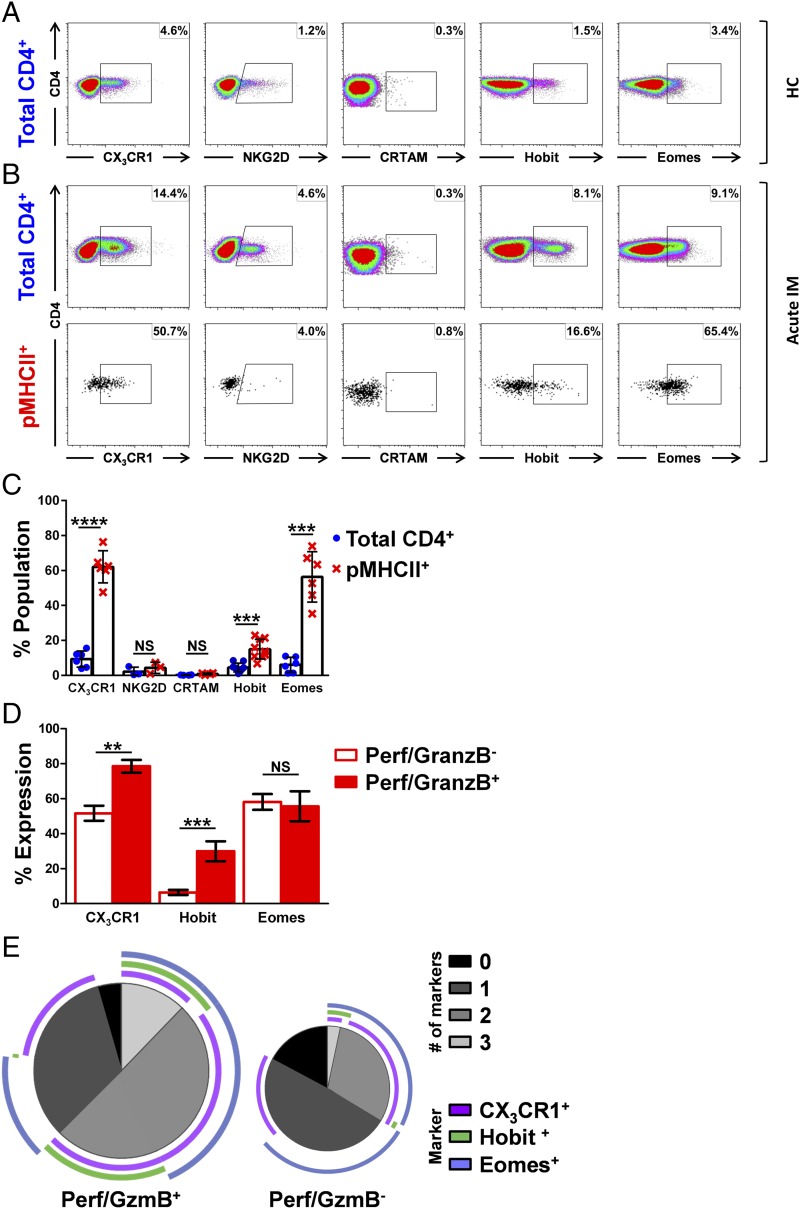
Acutely generated EBV-specific CD4-CTLs are transcriptionally distinct from classically defined virus-specific CD4-CTLs. (**A**) Expression of CX_3_CR1, NKG2D, CRTAM, Hobit, and Eomes in the total CD4^+^ T cell pool from a representative healthy carrier (HC). (**B**) Expression of CX_3_CR1, NKG2D, CRTAM, Hobit, and Eomes in the total CD4^+^ (top panels) and DR7/EBNA2_275–296_ tetramer^+^ CD4^+^ T cell populations (bottom panels) from a representative patient with IM. (**C**) Summary of CX_3_CR1, NKG2D, CRTAM, Hobit, and Eomes expression in total CD4^+^ (blue) and EBV-specific pMHCII tetramer^+^ CD4^+^ T cell populations (red) from patients with IM (*n* = 6). (**D**) Summary of CX_3_CR1, Hobit, and Eomes expression in Perf/GzmB^+^ (filled) and Perf/GzmB^−^ pMHCII tetramer^+^ CD4^+^ T cell populations (unfilled) from patients with IM (*n* = 6). (**E**) SPICE plots illustrating the expression profiles of CX_3_CR1, Hobit, and Eomes among Perf/GzmB^+^ and Perf/GzmB^−^ pMHCII tetramer^+^ CD4^+^ T cells from patients with IM (*n* = 6). Image sizes are scaled to depict the distribution of Perf/GzmB^+^ and Perf/GzmB^−^ events among pMHCII tetramer^+^ CD4^+^ T cells. Each pie chart segment displays the fraction of cells expressing the number of markers indicated in the key. Arcs show the distribution of each individual marker. (C–E) Error bars indicate means ± SD. Significance was determined by unpaired Student *t* test with Welch correction (c) and the Mann–Whitney U test for (d). **p < 0.01, ***p < 0.001, ****p < 0.0001. NS, not significant.

CD4-CTLs have to date been almost exclusively associated with late differentiated T_EMRA_ CD4^+^ cells that accumulate during viral persistence. In this context, several cellular markers have been associated with the development or function of CD4-CTLs, including the transcription factors Eomes (17, 39, 40) and Hobit (11), the surface receptors NKG2D (41, 42) and CX_3_CR1 (17, 43), and the class I–restricted T cell–associated molecule CRTAM (44). We therefore asked whether these markers were similarly expressed by the EBV-specific CD4-CTLs induced by acute infection. Initial experiments assessed expression in total CD4^+^ and pMHCII tetramer^+^ T cells. In a representative healthy carrier, each marker was expressed by 1–5% of total CD4^+^ T cells, with the exception of CRTAM (Fig. 4A). However, expression of all five markers was negligible on memory EBV-specific tetramer^+^ CD4^+^ T cells in all healthy carriers (*n* = 15; data not shown). Substantially higher frequencies of total CD4^+^ T cells expressed CX_3_CR1, NKG2D, Hobit, and Eomes in a representative patient with IM (Fig. 4B, top panels), and expression of CX_3_CR1, Hobit, and Eomes was further increased among pMHCII tetramer^+^ CD4^+^ T cells in the same donor (Fig. 4B, bottom panels). A similar pattern was observed across all patients with acute IM (Fig. 4C, *n* = 6). Accordingly, significantly higher expression of CX_3_CR1, Hobit, and Eomes but not NKG2D or CRTAM, was detected among EBV-specific CD4^+^ T cells compared with total CD4^+^ T cells (mean 62.0% versus mean 9.4% for CX_3_CR1, respectively, *p* < 0.0001; mean 15.0% versus mean 4.6% for Hobit, respectively, *p* < 0.001; mean 56.4% versus mean 6.1% for Eomes, respectively, *p* < 0.001).

Subsequent analyses of CX_3_CR1, Hobit, and Eomes expression among the Perf/GzmB^+^ and Perf/GzmB^−^ EBV-specific CD4^+^ T cells suggested that, during acute IM, EBV-specific CD4-CTLs differ in their transcriptional program from classically reported CD4-CTLs in other persistent viral infections. Thus, no single marker was expressed by all Perf/GzmB^+^ EBV pMHCII tetramer^+^ CD4^+^ T cells, and CX_3_CR1 and Eomes were commonly expressed by pMHCII tetramer^+^ CD4^+^ T cells lacking Perf/GzmB (Fig. 4D). Furthermore, no combination of these markers was exclusively associated with cytotoxic potential (Fig. 4E). Although the majority of Perf/GzmB^+^ pMHCII tetramer^+^ CD4^+^ T cells expressed CX_3_CR1 with Hobit and/or Eomes (Fig. 4E, left panel), similar combinatorial profiles were detected among Perf/GzmB^−^ pMHCII tetramer^+^ CD4^+^ T cells (Fig. 4E, right panel).

### Clonal evolution of EBV-specific CD4^+^ T cells

To determine if changes in functionality over time were associated with changes in clonal composition, we performed a longitudinal analysis of clonotype use among CD4^+^ T cell populations. We focused our efforts on the EBNA2_276–295_ epitope, which can be presented by DR7 or HLA-DRB3*02:02 (DR52b) (23), and against which expanded populations of CD4^+^ T cells have been observed during primary EBV infection (22).

**FIGURE 5.**
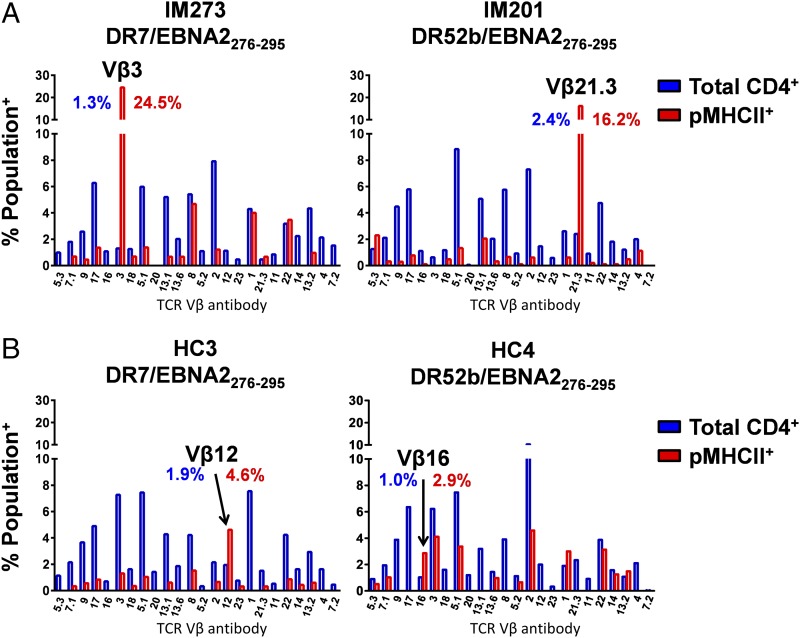
EBV-specific CD4^+^ T cells display biased TCR Vβ use in patients with IM. (**A** and **B**) CD4-enriched PBMCs from patients with IM (A) and healthy carriers (HC) (B) were stained with the indicated pMHCII tetramers and analyzed by flow cytometry for surface expression of defined TCR Vβ segments. Clonograms show the distribution of specific TCR Vβ segments among total CD4^+^ (blue) and EBV-specific pMHCII tetramer^+^ CD4^+^ T cells (red). The dominant TCR Vβ segment is highlighted in each case.

Initially, we used a commercially available Ab kit to examine TCR Vβ expression on the surface of DR7/EBNA2_276–295_–specific or DR52b/EBNA2_276–295_–specific CD4^+^ T cells. Successful dual staining of epitope-specific CD4^+^ T cell clones of known TCR Vβ usage showed that there was no detectable competition between Vβ-specific Abs and pMHCII tetramers for the TCR (Supplemental Fig. 3). Representative clonograms for DR7/EBNA2_276–295_–specific (donor IM273) and DR52b/EBNA2_276–295_–specific CD4^+^ T cells (donor IM201) are shown in Fig. 5A, annotated using the nomenclature of Wei et al. (45). In each of these patients with IM, the expressed TCR Vβ repertoire differed markedly between the EBV-specific CD4^+^ T cell population and the total CD4^+^ T cell pool. Repertoire bias was also observed in the EBV-specific CD4^+^ T cell populations. In donor IM273, 24.5% of DR7/EBNA2_276–295_–specific CD4^+^ T cells expressed Vβ3 compared with only 1.3% of the total CD4^+^ T cell pool, whereas in donor IM201, 16.2% of DR52b/EBNA2_276–295_–specific CD4^+^ T cells expressed Vβ21.3 compared with only 2.4% of the total CD4^+^ T cell pool. In contrast, the corresponding EBV-specific CD4^+^ T cell repertoire profiles in healthy carriers were more evenly distributed overall and incorporated only modest Vβ-defined expansions (Fig. 5B).

**FIGURE 6.**
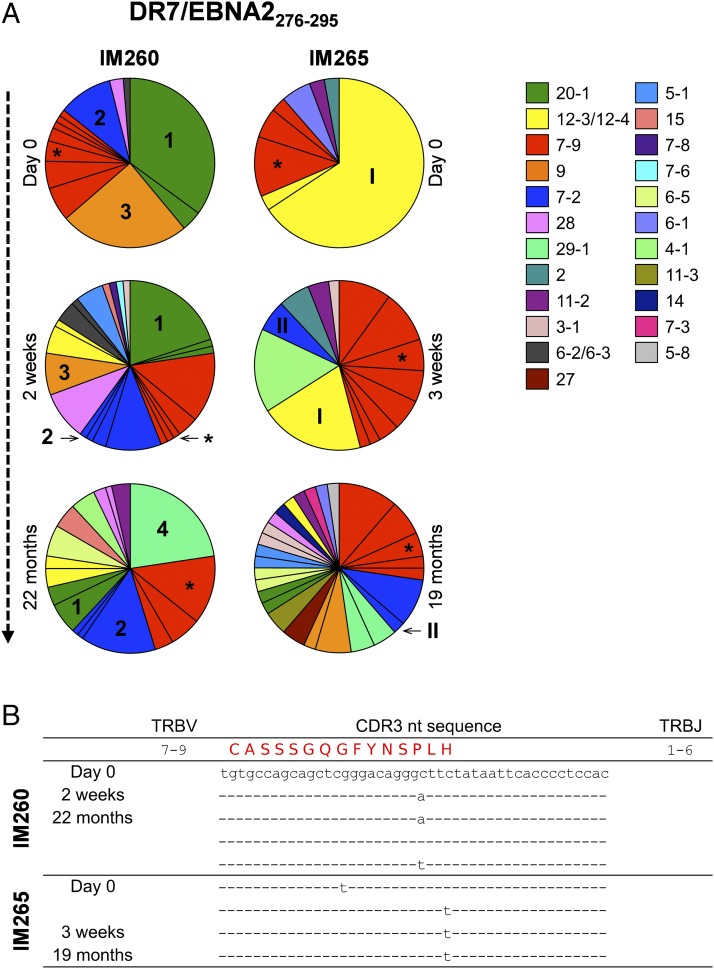
Primary EBV infection induces oligoclonal expansions of Ag-specific CD4^+^ T cells. (**A**) Serial analysis of expressed *TRB* gene transcripts in EBV-specific CD4^+^ T cell populations from patients with IM. Each pie chart segment denotes a unique clonotype. Dominant clonotypes are indicated as 1–4 in donor IM260, and I and II in donor IM265. A public sequence is indicated with an asterisk. (**B**) Nucleotide sequences encoding the public amino acid sequence.

Subsequently, we used an unbiased molecular approach to characterize all expressed *TRB* gene rearrangements in EBV-specific CD4^+^ T cell populations (31). Data were annotated using the International ImMunoGeneTics nomenclature (33). We first compared the repertoires of DR7/EBNA2_276–295_–specific CD4^+^ T cells in two patients with IM (donor IM260 and donor IM265). In accordance with the Vβ expression data (Fig. 5A), preferential *TRBV* gene use was apparent in both of these EBV-specific CD4^+^ T cell populations (Fig. 6, top panel). The DR7/EBNA2_276–295_–specific CD4^+^ T cell population in donor IM260 favored *TRBV20-1* (39%), whereas the DR7/EBNA2_276–295_–specific CD4^+^ T cell population in donor IM265 favored *TRBV12-3* (68%). A single dominant clonotype was responsible for the observed *TRBV* gene bias in each case. We then performed similar analyses using serial samples collected from the same donors at 2–3 wk and >18 mo after diagnosis. There was a progressive contraction of the initially dominant clonotypes in both donors (clone 1 and clone 3 in donor IM260 and clone I in donor IM265), and new clonotypes emerged over time, some of which became dominant (clone 4 in donor IM260 and clone II in donor IM265). Interestingly, a public *TRBV7-9*^+^ DR7/EBNA2_276–295_–specific clonotype (*) identified during acute infection was detected at all time points in both donors, demonstrating persistence into long-term memory. Although identical at the amino acid level, the corresponding CDR3β loops were differentially encoded at the nucleotide level (Fig. 6B), in line with the principles of convergent recombination (46, 47).

### Expression of cytotoxic proteins in EBV-specific CD4^+^ T cells is associated with activation

**FIGURE 7.**
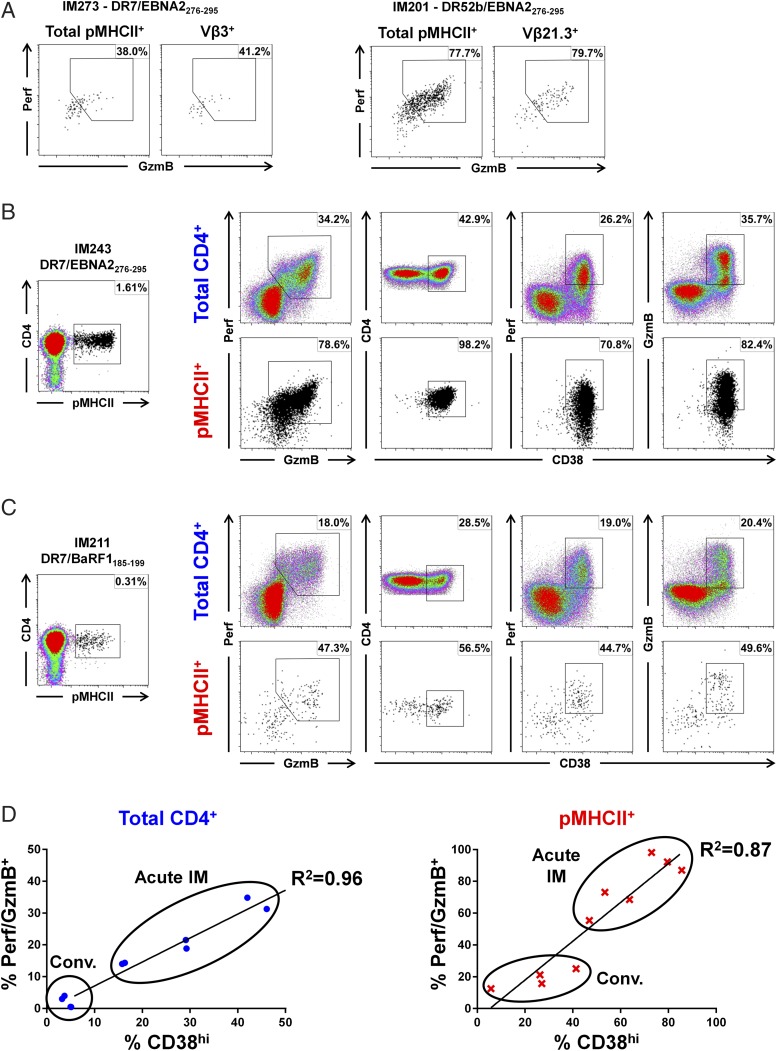
Expression of cytotoxic proteins in EBV-specific CD4^+^ T cells is associated with cellular activation. (**A**) Perf/GzmB expression among total pMHCII tetramer^+^ CD4^+^ (left plots) and dominant TCR Vβ^+^ pMHCII tetramer^+^ CD4^+^ T cells (right plots) from the donors shown in Fig. 5A. (**B** and **C**) CD4-enriched PBMCs from two representative patients with IM were stained with the relevant pMHCII tetramers (left plots) and analyzed by flow cytometry for expression of Perf/GzmB and CD38 (right plots) in the total CD4^+^ (top panels) and pMHCII tetramer^+^ CD4^+^ T cell populations (bottom panels). (**D**) Correlation between Perf/GzmB expression and CD38^hi^ events among total CD4^+^ (left plot, *p* < 0.0001) and pMHCII tetramer^+^ CD4^+^ T cell populations (right plot, *p* < 0.0001) from patients with acute and convalescent IM. Significance was determined using the Pearson correlation.

Given that the observations that Perf/GzmB and CX3CR1/Hobit expression were never universal within individual pMHCII tetramer^+^ CD4^+^ T cell populations (Fig. 3G), we assessed whether cytotoxicity was associated with dominant Vβ-defined expansions in patients with IM. However, there were no obvious associations between Perf/GzmB expression and TCR Vβ use among DR7/EBNA2_276–295_–specific CD4^+^ T cells in donor IM273 or DR52b/EBNA2_276–295_–specific CD4^+^ T cells in donor IM201 (Fig. 7A). Rather, Perf/GzmB expression was heterogeneous among Vβ-defined pMHCII tetramer^+^ CD4^+^ T cells and reflected that among the total pMHCII tetramer^+^ CD4^+^ T cell population. Importantly, however, these data suggest that individual clonotypes can comprise cells with different functional capacities.

A search for other correlates of cytotoxic potential revealed that Perf/GzmB expression was strongly associated with cellular activation in primary EBV infection (Fig. 7B–D). Within the total CD4^+^ T cell pool of donor IM243, Perf/GzmB expression was almost exclusively limited to cells expressing high levels of CD38^hi^ (Fig. 7B, top panel). Accordingly, in the heavily expanded pMHCII tetramer^+^ population, in which CD38^hi^ was almost ubiquitously expressed, the vast majority of cells were positive for Perf/GzmB (Fig. 7B, bottom panel). In donor IM211, in which the lower magnitude of the pMHCII tetramer^+^ population and lower overall frequencies of Perf/GzmB^+^ and CD38^hi^ events in the total CD4^+^ T cell pool indicated blood donation later after disease onset (Fig. 7C, top panel), a lower frequency of EBV-specific CD4^+^ T cells were Perf/GzmB^+^ (Fig. 7C, bottom panel). Importantly, however, expression of cytotoxic proteins was limited to those cells expressing CD38^hi^. The concatenated data from all patients with acute or convalescent IM (Fig. 7D) show a strong correlation between the percentage frequency of Perf/GzmB^+^ events and the percentage frequency of CD38^hi^ events among both total CD4^+^ T cells (*R*^2^ = 0.96, *p* > 0.0001) and EBV-specific pMHCII tetramer^+^ CD4^+^ T cells (*R*^2^ = 0.87, *p* > 0.0001). Together, these data demonstrate a clear association between cellular activation and the cytotoxic potential of EBV-specific CD4-CTLs.

## Discussion

The global CD4^+^ T cell pool comprises functionally diverse subsets characterized by the expression of distinct cytokines and transcription factors (3). However, the clonotypic architecture and functional properties of virus-specific CD4^+^ T cell populations remain poorly defined. We addressed these issues in the context of EBV infection, which elicits an array of cellular immune responses (20), including Ag-specific CD4^+^ T cell expansions amenable to analysis with pMHCII tetramers (22).

Using ex vivo peptide stimulation assays, we found that pMHCII tetramer^+^ CD4^+^ T cells produced IFN-γ, TNF-α, and IL-2 during persistent infection, consistent with a T_H_1-like profile regulated by T-bet (3) and also with previous in vitro studies implicating polyfunctional CD4^+^ T cells in the long-term control of EBV (14, 48, 49). Importantly, effector cytokines were also produced ex vivo in response to physiological levels of Ag presented on the surface of virus-infected B cells. Direct MHCII-restricted recognition of EBV-infected LCLs by CD4^+^ T cells has only previously been demonstrated in vitro (23–29), always in cultured or expanded cells. Such ex vivo reactivity in the absence of prior activation is an important indicator of direct effector functionality in vivo. Of note, LCL stimulation induced fewer functions per cell than peptide stimulation, potentially reflecting differences in cytokine production among epitope specificities within the broadly targeted CD4^+^ T cell response (20, 50, 51) and/or the delivery of a weaker antigenic stimulus (52). Nevertheless, in both settings, the cytokine response was dominated by TNF-α in healthy carriers, as reported for CD4^+^ T cells targeting viral epitopes in persistent CMV or HIV infection (40, 43).

During primary EBV infection, significantly greater percentages of circulating CD4^+^ T cells responded directly to ex vivo LCL stimulation. However, the responding cells in patients with IM typically produced fewer cytokines than LCL-responsive cells in healthy carriers, and the overall profile was dominated by IFN-γ. Increases in polyfunctionality in CD4^+^ T cells over time have similarly been observed during persistent CMV infection (53) and during primary EBV infection in children (14), potentially reflecting progressive differentiation and/or preferential selection of cognate CD4^+^ T cells bearing high-affinity TCRs (52, 53).

Ex vivo LCL challenge also induced degranulation among CD4^+^ T cells in patients with IM. Classically, human CD4-CTLs have been described in vivo almost exclusively in the setting of persistent viral infections, including CMV, dengue virus, and HIV (17, 43, 54). With regard to EBV, cytotoxic function in CD4^+^ T cells derived from healthy carriers has been reported but always following in vitro culture and expansion (24–26, 28). Surprisingly, upon ex vivo analysis, we found very little evidence of EBV-specific CD4-CTLs in healthy carriers. Rather, cytotoxic activity was limited almost exclusively to EBV-specific CD4^+^ T cells in patients with IM. Accordingly, most pMHCII tetramer^+^ CD4^+^ T cells expressed Perf/GzmB during acute infection, whereas very few pMHCII tetramer^+^ CD4^+^ T cells expressed Perf/GzmB during persistent infection. Moreover, we found that Perf/GzmB expression was significantly elevated in the total CD4^+^ T cell pool during acute EBV infection, consistent with previous indications of raised Perf in CD4^+^ T cells at this time (19). Given the absence of bystander activation among coexistent influenza virus–specific CD4^+^ T cell populations, which has been reported previously in the context of viral or vaccine challenge (55–57), this observation suggests that the cumulative effect of broadly targeted EBV-specific CD4^+^ T cell responses is sufficient to impact the total CD4^+^ T cell pool (22). Interestingly, in the convalescent phase, Perf/GzmB expression remained elevated among pMHCII tetramer^+^ CD4^+^ T cells compared with the corresponding specificities in healthy carriers. This potentially reflects continued Ag exposure because ongoing lytic replication is known to persist in the throat for many months following primary infection (58, 59). The subsequent establishment of viral latency may limit antigenic drive in long-term persistent infection, which, in turn, may explain the relative lack of Perf/GzmB expression among EBV-specific CD4^+^ T cells in healthy carriers. Furthermore, differences in the differentiation status of CD4^+^ T cells specific for different viruses may impact their functional profiles during persistent infection. Thus, EBV-specific memory CD4^+^ T cells typically occupy the earlier-differentiated T_CM_ and T_EM_ pools (22, 60), contrasting with later-differentiated T_EMRA_ cells predominantly seen in other persistent viral infections in which CD4-CTLs are commonly seen (17, 40, 43). Accordingly, acquisition of Perf/GzmB–mediated cytotoxicity among memory CD4^+^ T cells has been shown to increase with progressive differentiation (4, 53), as observed during persistent CMV and HIV infection (19, 43).

Several cellular markers have been associated with the development and/or function of CD4-CTLs in the setting of persistent viral infection or cancer (11, 17, 40, 41, 43). Of these, the transcription factors Eomes, which is associated with cytotoxicity programs in CD8^+^ T cells (61), and Hobit, which is associated with tissue residency (62, 63), as well as the fractalkine receptor CX_3_CR1, which is involved in the adhesion and trafficking of lymphocytes (64), were all raised among Perf/GzmB^+^ pMHCII tetramer^+^ CD4^+^ T cells in patients with IM. However, no individual marker or combination of markers defined all Perf/GzmB^+^ EBV-specific CD4^+^ T cells. Although the correlation with Perf/GzmB expression may be prone to underestimation because of the prior release of cytotoxic molecules in vivo during ongoing infection, ∼5% of Perf/GzmB^+^ pMHCII tetramer^+^ CD4^+^ T cells lacked expression of Eomes, Hobit, and CX_3_CR1. Together, these data suggest that EBV-specific CD4-CTLs elicited by primary EBV infection are transcriptionally distinct from classical CD4-CTLs described in persistent viral infection. Thus, another pathway must drive their Perf/GzmB expression, potentially involving other transcription factors and/or regulatory proteins (4, 65, 66).

We next investigated whether cytotoxic capacity in EBV-specific CD4^+^ T cells in primary EBV infection was associated with dominant Vβ-defined expansions. Oligoclonal expansions of EBV-specific CD8^+^ T cells, but not EBV-specific CD4^+^ T cells, have been detected previously in the setting of IM (67, 68). However, these early studies were restricted to low-definition technologies in total CD4^+^ T cell pools. In this study, using pMHCII tetramers, we found marked preferences for certain TCR Vβ-chains among EBV epitope-specific CD4^+^ T cell populations, which reflected dramatic expansions of highly dominant clonotypes in patients with IM. In addition, distinct *TRBV* biases within the same Ag specificities were noted across individuals, and a public clonotype was detected at low frequency, in line with previous studies of EBV epitope-specific CD8^+^ T cells (69). Moreover, the dominant clonotypes persisted into convalescence, albeit with a degree of contraction, indicating selection into the memory pool (70). Newly recruited clonotypes also emerged over time, as reported previously for epitope-specific CD8^+^ T cell populations in CMV infection (71). Of note, preferential use of *TRBV7-9* among DR7/EBNA2_276–295_–specific CD4^+^ T cells was observed at all time points across individuals, suggesting an important germline-encoded contribution to Ag recognition (72, 73).

Importantly, there was no evidence to suggest preferential expression of Perf/GzmB among Vβ-defined pMHCII tetramer^+^ CD4^+^ T cells in patients with IM. This observation aligns with a similar lack of association between TCR use and function among CMV-specific CD4^+^ T cells (53) and suggests that individual clonotypes can exist in different functional states (70). Instead, we found a strong correlation between Perf/GzmB expression and cellular activation, defined using the surrogate marker CD38. The association of activation and cytotoxic capacity held true in convalescence, in which only those EBV-specific CD4^+^ T cells that remained activated expressed Perf/GzmB. High levels of activated pMHCII tetramer^+^ CD4^+^ T cells during primary EBV infection is consistent with previous reports of increased CD38 expression in CD4^+^ T cells in primary HIV infection and following vaccinia virus vaccination (15, 74). However, it is yet unknown if the activated CD4^+^ T cells induced in these settings express Perf/GzmB; a recent report of GzmB transcripts in virus-specific CD4^+^ T cells elicited during primary CMV infection suggests that this warrants further investigation (11). The acute wave of activated EBV-specific CD4-CTLs in primary infection markedly contrasts with classically reported CD4-CTLs, which have long been considered a feature of viral persistence and terminal differentiation in the setting of other viral infections (4, 17, 40, 43). How the acutely generated EBV-specific CD4-CTLs are regulated at the transcriptional level in relation to chronically maintained CD4-CTLs remains to be determined.

The lack of ex vivo cytotoxic potential among EBV-specific memory CD4^+^ T cells contrasts with in vitro studies, which have shown that EBV-specific CD4^+^ T cell lines and clones derived from healthy carriers almost invariably express Perf/GzmB and exhibit direct cytotoxicity against EBV-infected B cells (23–26, 28, 29). Acquisition of cytotoxic functions during in vitro stimulation and culture does not appear to result from progressive cellular differentiation because EBV-specific CD4^+^ T cell clones retain an effector CCR7^−^CD45RA^−^ phenotype (24). It therefore seems likely that Perf/GzmB expression can be induced in EBV-specific memory CD4^+^ T cells upon Ag encounter, consistent with an intimate association between activation and cytotoxicity in the CD4^+^ T cell lineage. However, possible rapid expansion of a small minority of high-affinity Perf/GzmB^+^ memory CD4^+^ T cells that are retained in healthy carriers cannot be discounted.

In summary, we have shown that primary EBV infection elicits oligoclonal populations of highly activated and directly cytotoxic virus-specific CD4^+^ T cells with a T_H_1-like functional profile that respond immediately to ex vivo challenge with autologous LCLs. These findings suggest that activated CD4-CTLs have the potential to eliminate virus-transformed B cells in vivo, potentially informing the rational development of novel vaccines designed to combat EBV-associated diseases.

## Supplementary Material

Data Supplement
