# Supplementary material for: Primary EBV Infection Induces an Acute Wave of Activated Antigen-Specific Cytotoxic CD4+ T Cells
Source: J Immunol. 2019 Jul 15;203(5):1276–87. doi: 10.4049/jimmunol.1900377 (PMC6697742; doi:10.4049/jimmunol.1900377)
Supplement: Data Supplement [file JI_1900377.zip › JI_1900377_Supplemental_Figures_1.pdf]

Supplementary Figure 1

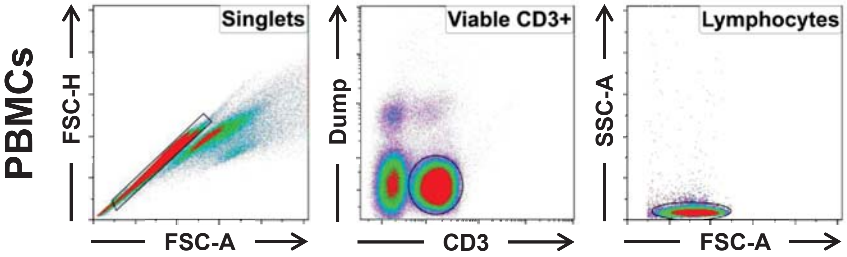

Supplementary Figure 1. Gating strategy for flow cytometric analyses. Single cells were gated in a forward scatter-height (FSC-H) *versus* forward scatter-area (FSC-A) plot, and a single dump channel was used to exclude dead cells, $CD14^+$ events, and $CD19^+$ events. Viable $CD3^+$ cells were then gated in a side scatter-area (SSC-A) *versus* FSC-A plot.

Supplementary Figure 2

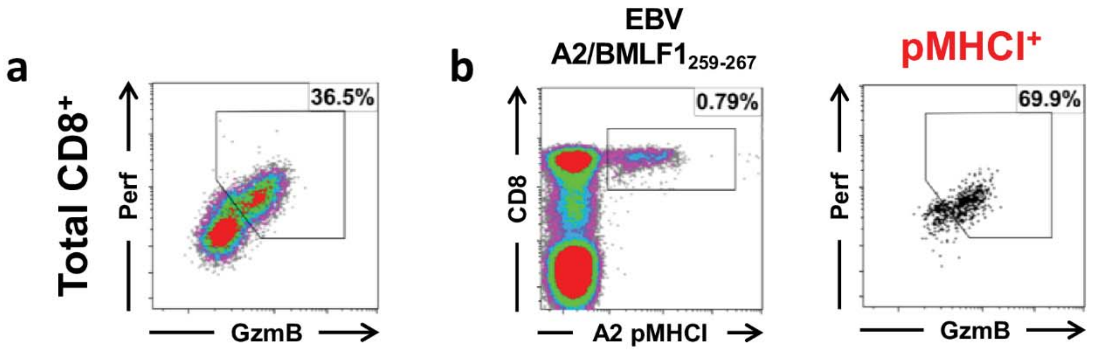

Supplementary Figure 2. Optimization of the gating strategy for detection of Perf/GzmB via analysis of an EBV-specific CD8$^+$ memory T cell population. (**a**–**b**) Whole PBMCs from an A2$^+$ healthy carrier were stained with A2/BMLF1$_{259\text{-}267}$ tetramer and analyzed by flow cytometry for intracellular expression of Perf/GzmB. (**a**) Perf/GzmB expression in the total CD8$^+$ T cell pool. (**b**) A2/BMLF1$_{259\text{-}267}$ tetramer staining of CD3$^+$ T cells (top panel), and Perf/GzmB expression among pMHCI tetramer$^+$ CD8$^+$ T cells (bottom panel).

Supplementary Figure 3

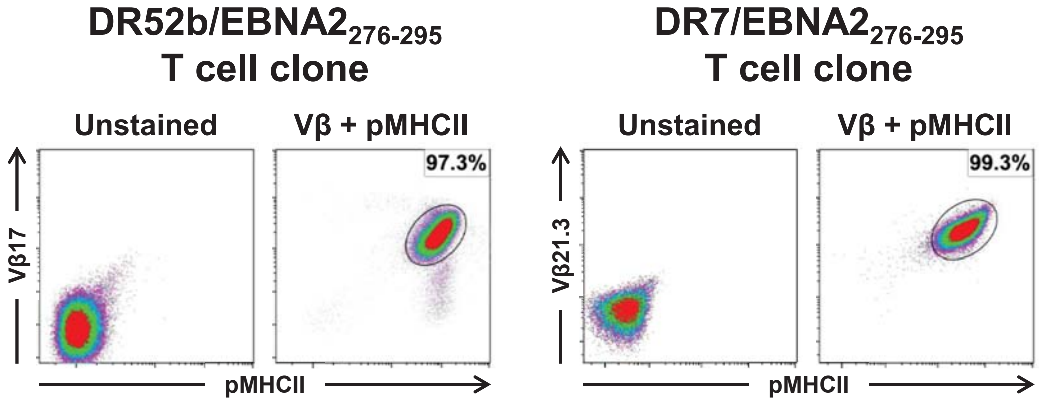

Supplementary Figure 3. Combinatorial staining with TCR Vβ-specific antibodies and pMHCII tetramers. EBNA2$_{276\text{-}295}$-specific CD4$^+$ T cell clones were stained sequentially with TCR Vβ-specific antibodies matching the expressed TCRs and pMHCII tetramers matching the corresponding restriction elements (DR52b, left panel; DR7, right panel).
